# Early Liver Dysfunction in Patients With Intra-Abdominal Infections

**DOI:** 10.1097/MD.0000000000001782

**Published:** 2015-10-23

**Authors:** Kun Guo, Jianan Ren, Gefei Wang, Guosheng Gu, Guanwei Li, Xiuwen Wu, Jun Chen, Huajian Ren, Zhiwu Hong, Lei Wu, Guopu Chen, Deng Youming, Jieshou Li

**Affiliations:** From the Department of General Surgery, Jinling Hospital, Medical School of Nanjing University, Nanjing, China.

## Abstract

Liver dysfunction is commonly seen in patients with severe sepsis; however, few studies were reported in intra-abdominal infections (IAIs). This study was performed to assess the risk factors for early liver dysfunction (ELD) in patients with IAIs and to determine the effects of ELD on outcomes of these patients.

From January 2011 to November 2014, a retrospective study that screened 421 patients with IAIs was performed. ELD was defined as an increase in serum total bilirubin (TB) >2 mg/dL or aminotransferases levels greater than twice the normal value within 48 hours after IAIs’ onset. Patients with pre-existing liver disease or major hepatobiliary injury were excluded. Risk factors for ELD and outcomes were compared by univariate and multivariate analyses. Subgroup analysis was performed for ELD patients within 24 to 48 hours.

Of 353 enrolled patients admitted with IAIs, 147 (41.6%) developed ELD. Significant independent risk factors for ELD were trauma (odds ratio [OR] 1.770, 95% confidential interval [CI] 1.126–2.783, *P* = 0.01) and abdominal compartment syndrome (ACS) (OR 3.199, 95% CI 1.184–8.640, *P* = 0.02). Successful source control <24 hours was shown to exert protection against ELD after 24 hours during IAIs (OR 0.193, 95% CI 0.091–0.409, *P* < 0.001). ELD was associated with significantly worse outcomes, including longer ICU length of stay and higher in-hospital mortality. Multivariate analysis also showed that development of ELD was a predisposing factor of mortality in IAIs patients (*P* < 0.001).

ELD was a common complication in patients with IAIs associated with worse outcomes. Trauma and ACS were relevant risk factors. Early successful source control appeared to be an important method to prevent and/or reduce ELD in patients with IAIs.

## INTRODUCTION

The liver as an important immune and metabolic organ is closely linked to several major biological functions such as synthesis, detoxification, inflammatory response, and blood clotting etc. In critically ill patients, the development of liver dysfunction (LD) complicates the clinical picture and poses a significant clinical challenge both in diagnostic evaluation and management.^1,2^ LD has been considered a prominent feature of the multiple organ dysfunction syndrome (MODS) and has been defined predominantly by hyperbilirubinemia and clinical jaundice.^3,4^

Infections, hemodynamic instability, renal insufficiency, hepatotoxic drugs, multiple blood transfusions, and/or total parenteral nutrition (TPN) administration are some of the potential causes of jaundice.^5–9^ A number of studies have shown that LD contributes to poor outcomes in critically ill patients. Notably early LD (ELD) is associated with higher in-hospital mortality.^6,10,11^ These studies, however, were conducted in the setting of diverse populations of MODS patients with varying etiologies and may not be applicable to the patients with intra-abdominal infections (IAIs).

IAIs are an important cause of morbidity and mortality in the intensive care unit (ICU).^12,13^ Some authors have demonstrated LD was more frequently present in the peritonitis and trauma patients, in the absence of pre-existing liver disease.^7,14,15^ However, there are few detailed reports of its nature in patients with IAIs. Since strategies to solve LD remain limited, a timely and accurate identification of factors promoting LD may lead to prevention or attenuation of its consequences. Accordingly, the aim of this study was to investigate the risk factors for the development of ELD and to determine the effects of ELD on outcomes of these patients.

## METHODS

### Study Design

This was a retrospective study of patients with IAIs admitted to a surgical ICU in Jinling Hospital, China. All the enrolled patients had IAIs due to trauma or septic intra-abdominal complications following surgery. The study was conducted in accordance with the ethical principles of the Declaration of Helsinki (and subsequent revisions) and to the current norm for observational studies.

## PATIENTS

Consecutive patients older than 18 years who were diagnosed with IAIs between January 2011 and November 2014 were screened for inclusion. Patients who died or were discharged 48 hours or earlier after admission, or manifested acute liver failure, decompensated chronic liver disease, or primary hepatobiliary involvement on admission were excluded. Primary hepatobiliary involvement was defined as liver trauma, hepatitis, malignancy, and cholecystitis. For patients who were admitted more than once, only the first admission was evaluated. The blood routine and biochemical determination were assessed in all patients daily. The tested index for study purpose included total bilirubin (TB), alanine aminotransferase (ALT), aspartate aminotransferase (AST), alkaline phosphatase (ALP), and γ-glutamyl transpeptidase (GGT). The standard values of laboratory tests in our hospital were as follows: TB, 0.1–1 mg/dL; ALT, 2–50 U/L; AST, 2–50 U/L; GGT, <50 U/L; ALP, 30–120 U/L.

Sepsis and septic shock were diagnosed according to the criteria of the American College of Chest Physician/Society of Critical Care Medicine.^3^ SIRS was defined by ≥2 of the following conditions: temperature >38°C or <36°C; heart rate >90 beats/min; respiratory rate >20 breaths/min or PaCO2 <4.26 kPa; white blood cell count >12,000 or <4000 cells/mL (or >10% immature forms). Sepsis was defined as a systemic response to infection including the criteria of SIRS plus microbiological evidence of a focal infection and/or a positive blood culture. Septic shock was defined by the persistent presence of sepsis-induced tissue hypoperfusion refractory to adequate fluid resuscitation. Samples for microbiological cultures were collected routinely when the patients had fever (>38°C) and there was a clinical suspicion or evidence of infection.

### Data Collection and Definitions

The collected data included demographic data and comorbid diseases, causes of admission, APACHE II (Acute Physiology and Chronic Health Evaluation II) score, and sequential organ failure assessment (SOFA) (Sequential Organ Failure Assessment) score within 24 h following admission, the number of blood transfusions and positive bacterial cultures, the need for mechanical ventilation, and continuous renal replacement therapy (CRRT). Meanwhile, the records of percutaneous catheter drainage under color doppler ultrasound and open abdomen management were all registered. Additional data were obtained from the computerized hospital medical records.

ELD in patients with IAIs was defined according to the following criteria: serum TB levels >2 mg/dL or aminotransferases levels greater than twice the normal value within 48 hours after the onset of IAIs. Source control is critical for amelioration of IAIs. Hence we further selected those who developed ELD within 24 to 48 hours after admission, and investigated whether early successful source control (<24 hours) exerted protection against subsequent ELD (within 24–48 hours).

Intra-abdominal hypertension (IAH) was defined as a sustained or repeated pathologic elevation of the intra-abdominal pressure (IAP) ≥12 mmHg.^16^ Abdominal compartment syndrome (ACS) was defined as a sustained IAP ≥20 mmHg that is associated with new onset of organ dysfunction or failure.^16^

Successful source control was identified according to a clinician's assessment and established definition^17^: resolution of fever, oral temperature <37.5°C; resolution of leukocytosis, white blood cells <12.0 × 10^9^ cells/L, and absence of bands and immature neutrophil forms; resolution of physical findings of tenderness and rigidity and restoration of enteric function; no further operative or percutaneous intervention required.

All enrolled patients were treated according to the following principles: source control by surgical or percutaneous drainage; antibiotics therapy was initiated in all patients; other supporting treatments as needed, severe sepsis, and septic shock were managed by standard therapies.^12,18^ All patients with IAIs were followed until death or 60 days after discharge. We used in-hospital mortality rate as the primary outcome. Other outcomes including 60-day mortality, ICU lengths of stay (LOSs).

### Statistical Analyses

Statistical analyses were performed using SPSS software, version 16.0 (SPSS Inc, Chicago, IL) for Windows. Categorical data are expressed as numbers and/or percentages. Continuous variables are expressed as mean ± standard deviation (SD). Differences of continuous data between groups were compared using Student *t* test or repeated measures analysis of variance, and differences in categorical data were compared using Pearson *χ*^2^ test. The prognostic relevance of 2 different parameters was analyzed: variables associated with development of ELD and the prognostic value of ELD. All variables associated with ELD at the *P* < 0.20 level of risk in the binomial analysis were introduced in the first backward stepwise logistic regression model. Additionally, second model was constructed in a similar way, in-hospital mortality as the dependent variable, including all the variables yielding *P* < 0.20 by univariate analysis and those considered clinically relevant. A *P* value <0.05 was considered significant.

## RESULTS

**FIGURE 1:**
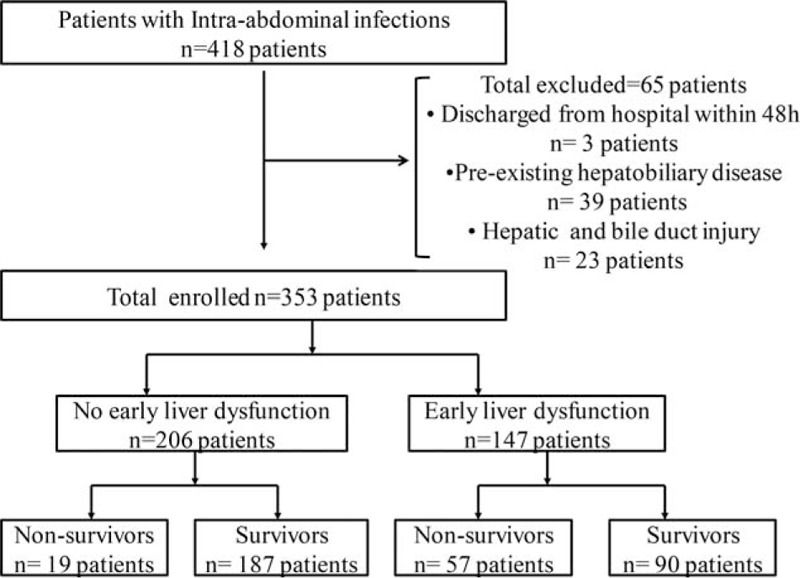
Flow of patients through the study.

Over the observation period from January 2011 to November 2014, a total of 418 consecutive patients met the registration criteria and were enrolled in the study. Among them, 3 patients were discharged from hospital within 48 hours. Thirty-nine patients were excluded from this study because of pre-existing liver disease (1 had liver abscess, 20 had cholecystitis and cholangitis, 18 had cirrhosis or viral hepatitis, hepatobiliary carcinoma) and 23 patients were excluded because of the presence of hepatobiliary injuries (3 patients had bile leakage, 9 had liver damage or portal vein injury, and 11 had severe operative bile duct injury or partial hepatectomy). A total of 353 patients were finally entered into the study. Figure 1 illustrates the enrollment and follow-up of study patients.

### Patients’ Characteristics

There were 147 (41.6%) patients who developed ELD. Baseline characteristics were presented in Table 1. The patients with ELD did not have significant differences in the age, body mass index (BMI), sepsis, positive bacterial cultures, and underlying diseases as compared with those without ELD. ELD patients had a male predominance (*P* = 0.65), and were more likely to have septic shock (*P* = 0.002), higher APACHE II score (*P* < 0.001), and SOFA score (*P* < 0.001). Patients with ELD suffered from trauma, IAP, and ACS more frequently. Further, a similar result was observed when the subgroup analysis was conducted between Non-ELD and ELD after 24 hours. Specifically, we found that successful source control <24 hours was significantly associated with non-ELD, when compared with ELD after 24 hours (85.4% vs 54.4%, *P* < 0.001).

**TABLE 1:**
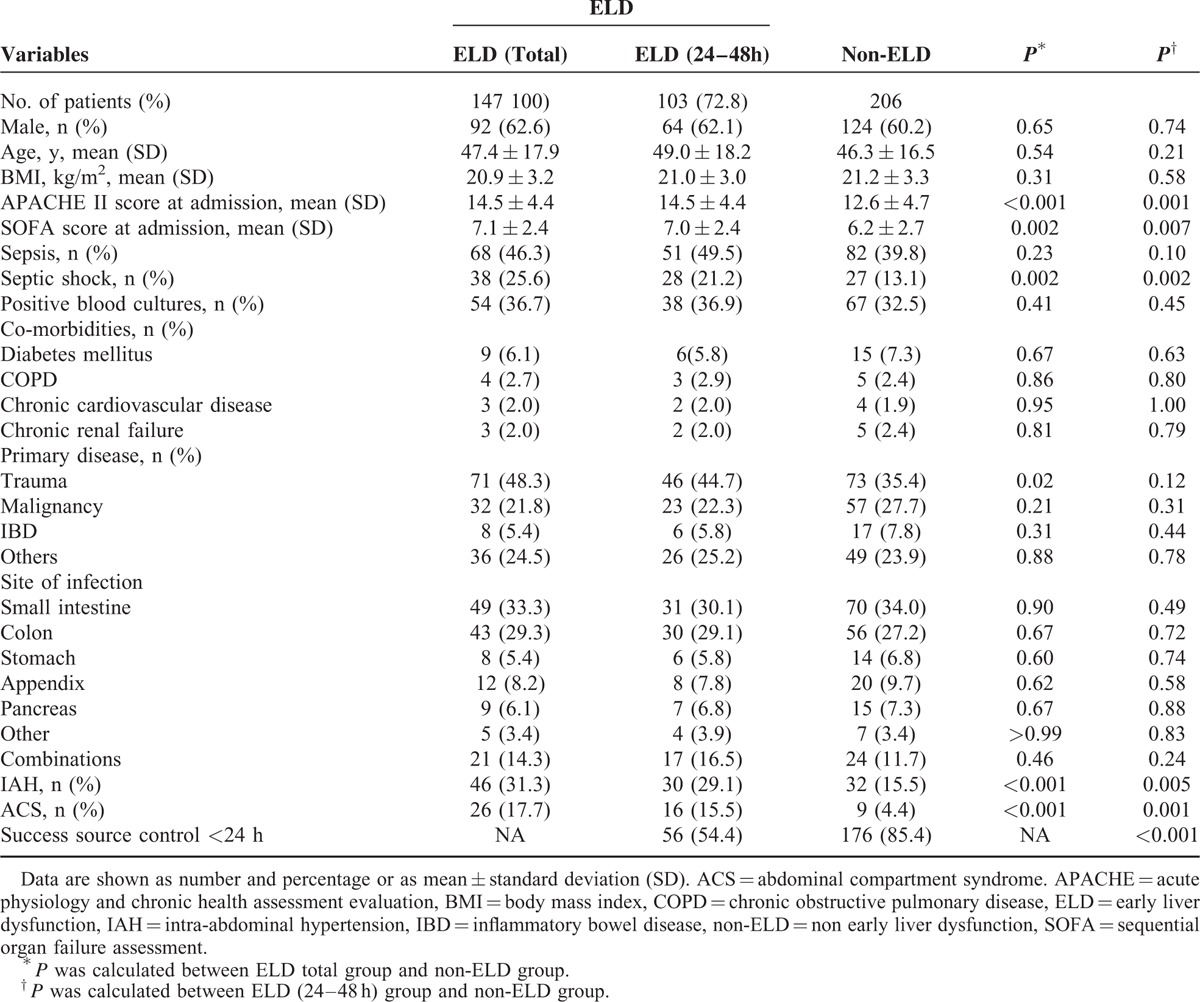
Comparison of Baseline Characteristics Between Groups With and Without Early Liver Dysfunction

### Independent Risk Factors of ELD

On multivariate analysis (Table 2), trauma (odds ratio [OR] 1.770, 95% confidential interval [CI] 1.126–2.783, *P* = 0.01) and ACS (OR 3.199, 95% CI 1.184–8.640, *P* = 0.02) were independent risk factors for ELD. Notably, patients with successful source control <24 hours were at a lower risk to develop ELD after 24 hours during IAIs (OR 0.193, 95% CI 0.091–0.409, *P* < 0.001). The rest of the variables analyzed, such as, severity of illness, septic shock, IAP, did not reach statistical significance in the logistical regression model.

**TABLE 2:**
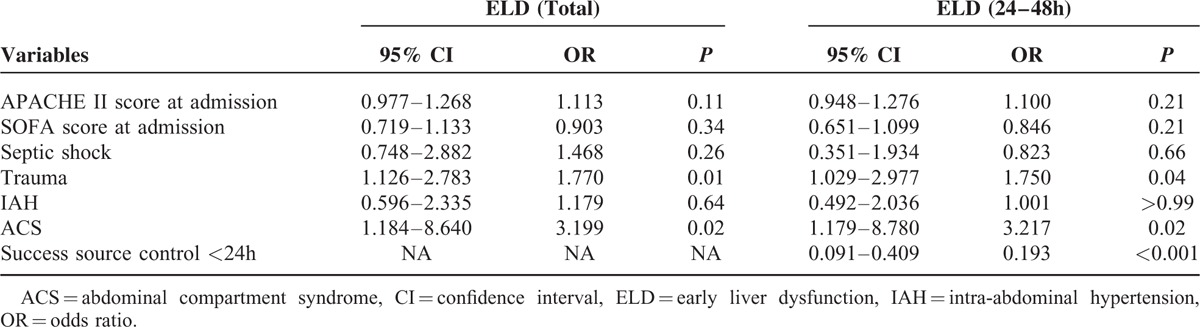
Stepwise Logistic Regression Analysis of the Variables for the Incidence of ELD in Patients With IAIs

### Comparison of Laboratory Characteristics

Among the 147 ELD patients, 142 (96.6%) patients had elevated serum TB levels. The elevated bilirubin level in plasma consists predominantly of conjugated bilirubin (date not shown). Only 16 (10.9%) patients had ALT levels>100 U/dL and 15 (10.2%) patients had ALT levels >100 U/dL (Table 3). We next examined the characteristics between the survivors and nonsurvivors in ELD group in terms of liver function parameters (Supplemental 1, http://links.lww.com/MD/A462). Most laboratory characteristics on ICU admission did not differ significantly between overall survivors and nonsurvivors in ELD group. ALP levels (*P* = 0.005) and TB levels were higher (*P* = 0.004) in survivors with ELD. In contrast, peak liver function parameters (TB, ALT, AST, ALP, and GGT) in patients of ELD were significantly higher in survivors compared with nonsurvivors.

**TABLE 3:**
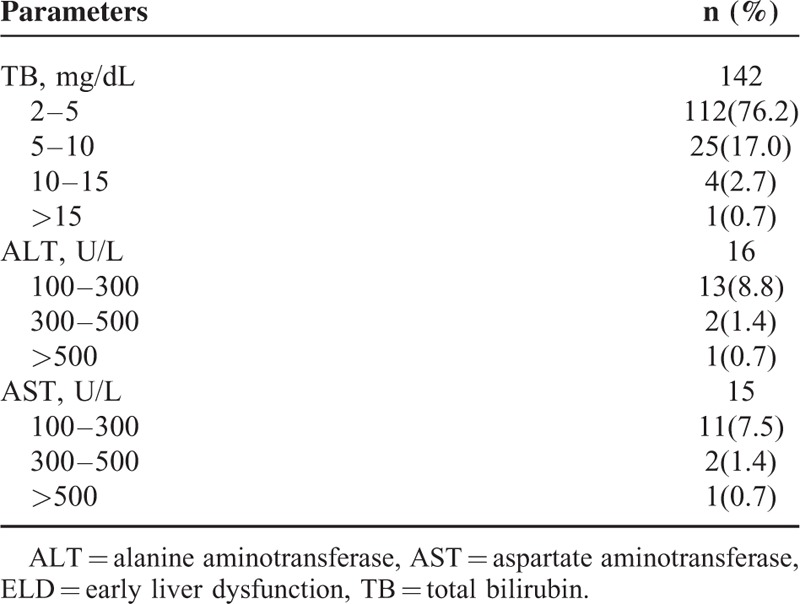
Severity of Abnormality of ELD

### Treatment in Hospital and Outcomes

As for the treatment, the percentage of patients who required open abdomen management (*P* < 0.001), percutaneous catheter drainage (*P* < 0.001), mechanical ventilation (*P* < 0.001), CRRT (*P* = 0.001), and blood transfusions (*P* = 0.002) were differed significantly in patients with or without ELD (Table 4). Patients with ELD had higher 60-day (*P* < 0.001) and in-patient mortality rates (*P* < 0.001). Likewise, ELD patients had significantly longer LOS ICU (*P* < 0.001) (Table 4). According to the logistic regression analysis, ELDs were found as independent risk factors for in-hospital mortality (OR 8.185, 95% CI 3.360–19.944, *P *< 0.001) (Table 5).

**TABLE 4:**
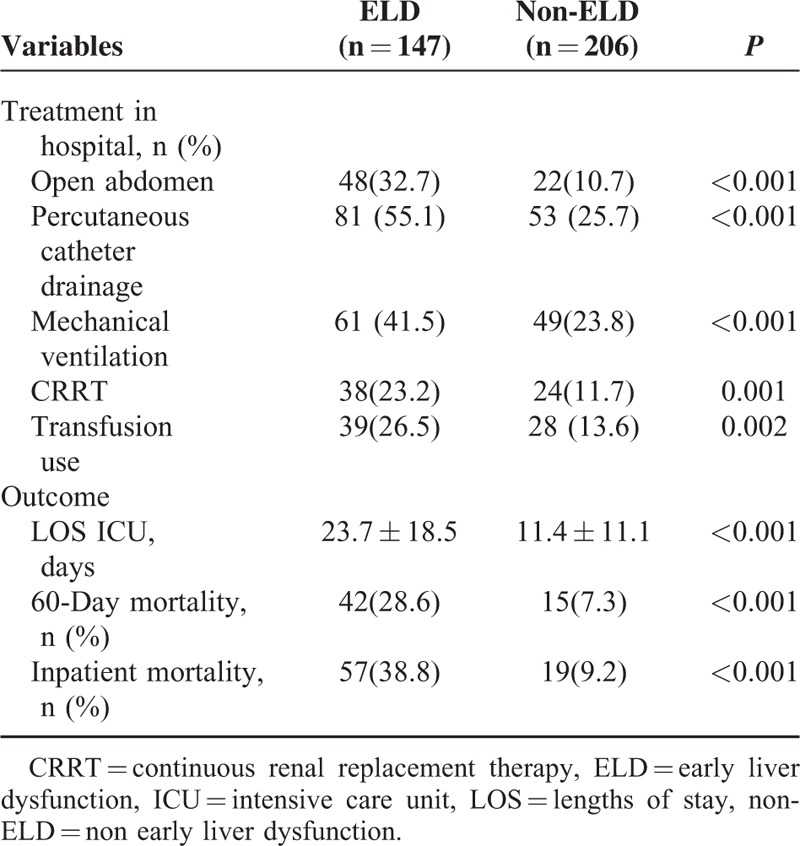
The Comparison of Treatment and Clinical Outcomes Between Patients With ELD and Non-ELD

**TABLE 5:**
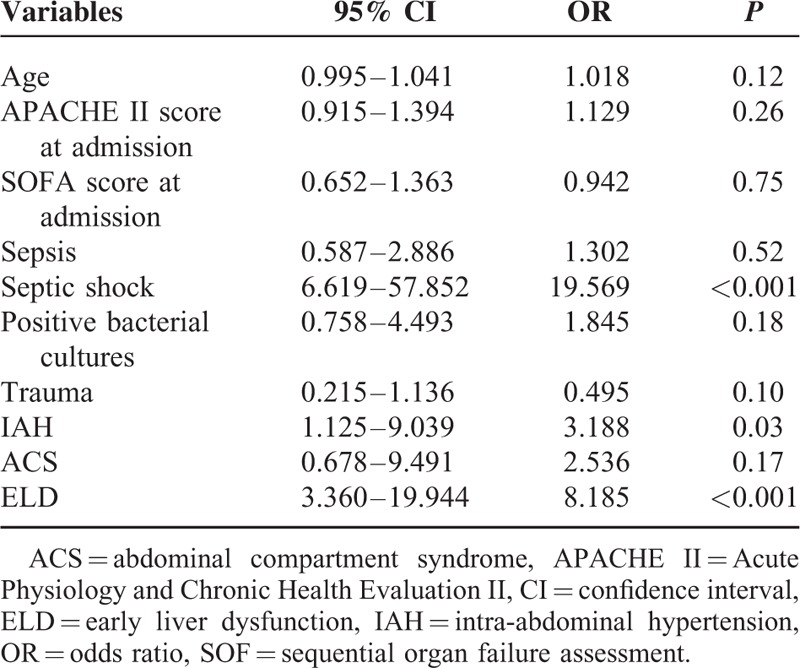
Stepwise Logistic Regression Analysis of the Variables for the In-Hospital Mortality

## DISCUSSION

Sepsis-associated LD is traditionally viewed as a late feature of critical illness indicated by jaundice and hyperbilirubinemia. However, recent studies have revealed LD as an early event in sepsis.^19–21^ In the present study, we observed 41.6% of IAIs patients who developed ELD, which was considerably higher than that of more heterogeneous patient populations reported by other investigators.^1,4–6^ Our result from a large study population indicated that ELD was associated with prolonged ICU and increased in-hospital mortality in patients with IAIs, which was consistent with other studies.^1,6^ ELD was a predictor of mortality in IAIs patients in association with other clinical factors. These data suggested that the development of ELD in patients with IAIs was a significant clinical event that had a measurable impact on outcome of the patients. Additionally, the present study revealed that there were significant differences between nonsurvivors and survivor patients with respect to serum TB and liver enzymes (AST, ALT), which is consistent with the pattern of liver cellular injury. Therefore, particular attention must be paid to the abnormal liver function, especially for the patients with continuous elevation of liver parameters.

Identifying the mechanisms underlying liver injury should aid in the development of effective therapies for ELD.^22,23^ Descriptive reports have suggested that the causes of LD were multiple—transfusion, TPN, infection, splanchnic ischemia, hepatotoxic medications—but both the pathogenesis and the clinical consequences were obscure.^5,6,9,23^ These factors are commonly encountered in patients with sepsis in the ICU. From our data, neither baseline APACHE II score nor septic shock was a risk factor for ELD. However, trauma and ACS were independently associated with development of ELD in our study. Interestingly, successful source control <24 hours was shown to exert protection against ELD.

Prior investigations have reported that abnormalities of hepatic function were found frequently in trauma patients, even in the absence of preexisting hepatobiliary disease.^14,24^ In the current study, our results suggested that trauma was a significant risk factor for ELD. In major trauma setting, ischemia/reperfusion liver injury caused by hypotension and hypoxemia after shock and resuscitation may play an important role in the development of ELD.^25,26^ In addition, under acute phase response, increased catabolism and insulin resistance triggered by systemic stress, leads to hypercholesterolemia and hyperglycemia. Lipid or glucose overload has direct inflammatory effects in the hepatocytes.^24,27^ Other causes include hemolysis after massive blood transfusion and hematomas resorption, which leads to bilirubin overload.^28^

Increased IAP causes regional hypoperfusion to all of the organs in the splanchnic bed.^29^ This effect might be most pronounced in the liver. Our study showed that ACS was an independent risk factor for development of ELD. Muftuoglu et al^30^ reported that liver function severely affected the onset of ACS and sepsis. The liver injury resulting from sepsis plus ACS is more severe than that resulting from either one independently. Hepatic arterial blood flow, portal venous blood flow, and indocyanine green plasma disappearance rate clearance were all decreased during ACS. Severe progressive reduction in mesenteric, arterial, portal, and microcirculatory blood flow had been shown with graded elevation in ACS.^31^

Source control seemed to be another independent predictor of ELD. In our study, patients achieving successful source control <24 hours after admission were at a lower risk to develop ELD after 24 hours (OR 0.193, 95% CI 0.091–0.409, *P* < 0.001). In patients with IAIs, the occurrence of ELD may be associated with a large amount of inflammatory mediators in the abdominal cavity, which is the originating infection. Intra-abdominal infection leads to impaired intestinal immunobarrier, increased intestinal permeability, bacterial overgrowth, and translocation of bacteria and/or bacterial products reach the liver via the portal vein. Bacteria and bacterial toxins directly disturb its normal synthetic, metabolic, excretory, and biotransformation functions, resulting in liver damage and jaundice.^5^ Furthermore, the liver is extremely important immune organ contains many important immune cells such as sinusoidal endothelial cells, hepatocytes, and stellate cells, especially Kupffer cells. Once activation, Kupffer cells produce a great variety of inflammatory mediators leading to sinusoidal endothelial cells impairment and bile duct cells secretion dysfunction, accompanied by hepatocellular retention of bilirubin, bile acids, and exogenous substances.^8,23,32^ Successful source control prevents amplification of inflammation, restores homeostasis within the abdominal cavity, and improves local and systemic immune responsiveness.^17^ Jimenez et al^33^ reported treatments focusing mainly on eradicating underlying infection and managing sepsis; source control remained the cornerstone of the management of IAIs. A delayed diagnosis and the initiation of source control significantly worsen the prognosis.^34,35^ Source control of infection plays a critical role in avoiding the occurrence and progress of LD.

We admit limitations in the present study. First, this was a retrospective study performed at a single tertiary-care medical center, which may result in selection bias to some extent. Second, because of the multifactor of the development of LD and the diagnosis of LD still remains imprecise, different LD patterns were not defined in our study. Third, we did not use a dynamic test, such as the plasma indocyanine green (ICG) disappearance rate (PDR_ICG_), to assess liver function. Finally, our study includes patient data of a surgical ICUs of IAIs. Accordingly, the conclusions drawn from this study might not necessarily be applicable to other surgical wards. However, to the best of our knowledge, our work is the very first study in this field and we believe our results in this issue may help the provision of future preventative strategies of ELD.

## CONCLUSION

In conclusion, our study demonstrated that ELD prolonged ICU stay and increased in-hospital mortality, which occurred in 41.6% of patients with IAIs. Trauma and ACS were risk factors for IAIs-related ELD. Early successful source control appeared to be an important method to prevent and/or reduce ELD in patients with IAIs.

## References

[1] Harbrecht BG Zenati MS Doyle HR Hepatic dysfunction increases length of stay and risk of death after injury. *J Trauma* 2002; 53:517–523. 12352490 10.1097/00005373-200209000-00020

[2] Jager B Drolz A Michl B Jaundice increases the rate of complications and one-year mortality in patients with hypoxic hepatitis. *Hepatology (Baltimore, Md)* 2012; 56:2297–2304. 10.1002/hep.25896 22706920

[3] Dellinger RP Levy MM Rhodes A Surviving Sepsis Campaign: international guidelines for management of severe sepsis and septic shock, 2012. *Intensive Care Med* 2013; 39:165–228. 23361625 10.1007/s00134-012-2769-8 PMC7095153

[4] Blanco J Muriel-Bombin A Sagredo V Incidence, organ dysfunction and mortality in severe sepsis: a Spanish multicentre study. *Crit Care (London, England)* 2008; 12:R158. 10.1186/cc7157 PMC2646323 19091069

[5] Kobashi H Toshimori J Yamamoto K Sepsis-associated liver injury: Incidence, classification and the clinical significance. *Hepatol Res* 2013; 43:255–266. 22971102 10.1111/j.1872-034X.2012.01069.x

[6] Kramer L Jordan B Druml W Incidence and prognosis of early hepatic dysfunction in critically ill patients-a prospective multicenter study. *Crit Care Med* 2007; 35:1099–1104. 17334250 10.1097/01.CCM.0000259462.97164.A0

[7] Labori KJ Bjornbeth BA Raeder MG Aetiology and prognostic implication of severe jaundice in surgical trauma patients. *Scand J Gastroenterol* 2003; 38:102–108. 12608472 10.1080/00365520310000519

[8] Nesseler N Launey Y Aninat C Clinical review: the liver in sepsis. *Crit Care (London, England)* 2012; 16:235. 10.1186/cc11381 PMC3682239 23134597

[9] Grau T Bonet A Rubio M Liver dysfunction associated with artificial nutrition in critically ill patients. *Crit Care (London, England)* 2007; 11:R10. 10.1186/cc5670 PMC2147066 17254321

[10] Kaffarnik MF Lock JF Vetter H Early diagnosis of sepsis-related hepatic dysfunction and its prognostic impact on survival: a prospective study with the LiMAx test. *Crit Care (London, England)* 2013; 17:R259. 10.1186/cc13089 PMC4057158 24172237

[11] Brun-Buisson C Meshaka P Pinton P EPISEPSIS: a reappraisal of the epidemiology and outcome of severe sepsis in French intensive care units. *Intens Care Med* 2004; 30:580–588. 10.1007/s00134-003-2121-4 14997295

[12] Solomkin JS Mazuski JE Bradley JS Diagnosis and management of complicated intra-abdominal infection in adults and children: guidelines by the Surgical Infection Society and the Infectious Diseases Society of America. *Clin Infect Dis* 2010; 50:133–164. 20034345 10.1086/649554

[13] De Waele J Lipman J Sakr Y Abdominal infections in the intensive care unit: characteristics, treatment and determinants of outcome. *BMC Infect Dis* 2014; 14:420. 25074742 10.1186/1471-2334-14-420 PMC4122779

[14] te Boekhorst T Urlus M Doesburg W Etiologic factors of jaundice in severely ill patients. A retrospective study in patients admitted to an intensive care unit with severe trauma or with septic intra-abdominal complications following surgery and without evidence of bile duct obstruction. *J Hepatol* 1988; 7:111–117. 3183348 10.1016/s0168-8278(88)80514-2

[15] Nishida T Fujita N Megawa T Postoperative hyperbilirubinemia after surgery for gastrointestinal perforation. *Surg Today* 2002; 32:679–684. 12181716 10.1007/s005950200126

[16] Field E Horst HM Rubinfeld IS Hyperbilirubinemia: a risk factor for infection in the surgical intensive care unit. *Am J Surg* 2008; 195:304–306.discussion 306-307. 18206848 10.1016/j.amjsurg.2007.12.010

[17] Solomkin JS Ristagno RL Das AF Source control review in clinical trials of anti-infective agents in complicated intra-abdominal infections. *Clin Infect Dis* 2013; 56:1765–1773. 23463643 10.1093/cid/cit128

[18] Dellinger RP Levy MM Rhodes A Surviving sepsis campaign: international guidelines for management of severe sepsis and septic shock: 2012. *Crit Care Med* 2013; 41:580–637. 23353941 10.1097/CCM.0b013e31827e83af

[19] Kortgen A Paxian M Werth M Prospective assessment of hepatic function and mechanisms of dysfunction in the critically ill. *Shock (Augusta, Ga )* 2009; 32:358–365. 10.1097/SHK.0b013e31819d8204 19197231

[20] Recknagel P Gonnert FA Westermann M Liver dysfunction and phosphatidylinositol-3-kinase signalling in early sepsis: experimental studies in rodent models of peritonitis. *PLoS Med* 2012; 9:e1001338. 23152722 10.1371/journal.pmed.1001338 PMC3496669

[21] Gonnert FA Recknagel P Hilger I Hepatic excretory function in sepsis: implications from biophotonic analysis of transcellular xenobiotic transport in a rodent model. *Crit Care (London, England)* 2013; 17:R67. 10.1186/cc12606 PMC4057165 23574754

[22] Chand N Sanyal AJ Sepsis-induced cholestasis. *Hepatology (Baltimore, Md )* 2007; 45:230–241. 10.1002/hep.21480 17187426

[23] Wu Z Han M Chen T Acute liver failure: mechanisms of immune-mediated liver injury. *Liver Int* 2010; 30:782–794. 20492514 10.1111/j.1478-3231.2010.02262.x

[24] Dahn MS Mitchell RA Lange MP Hepatic metabolic response to injury and sepsis. *Surgery* 1995; 117:520–530. 7740423 10.1016/s0039-6060(05)80251-x

[25] Wang P Hauptman JG Chaudry IH Hepatocellular dysfunction occurs early after hemorrhage and persists despite fluid resuscitation. *J Surg Res* 1990; 48:464–470. 2352422 10.1016/0022-4804(90)90014-s

[26] Champion HR Jones RT Trump BF Post-traumatic hepatic dysfunction as a major etiology in post-traumatic jaundice. *J Trauma* 1976; 16:650–657. 8649 10.1097/00005373-197608000-00010

[27] Grau T Bonet A Caloric intake and liver dysfunction in critically ill patients. *Curr Opin Clin Nutr Metab Care* 2009; 12:175–179. 19202389 10.1097/MCO.0b013e3283252f9e

[28] Labori KJ Raeder MG Diagnostic approach to the patient with jaundice following trauma. *Scand J Surg* 2004; 93:176–183. 15544071 10.1177/145749690409300302

[29] Carr JA Abdominal compartment syndrome: a decade of progress. *J Am Coll Surg* 2013; 216:135–146. 23062520 10.1016/j.jamcollsurg.2012.09.004

[30] Muftuoglu MA Aktekin A Ozdemir NC Liver injury in sepsis and abdominal compartment syndrome in rats. *Surg Today* 2006; 36:519–524. 16715421 10.1007/s00595-006-3196-7

[31] Cheatham ML Abdominal compartment syndrome: pathophysiology and definitions. *Scand J Trauma Resusc Emerg Med* 2009; 17:10. 19254364 10.1186/1757-7241-17-10 PMC2654860

[32] Bauer M Press AT Trauner M The liver in sepsis: patterns of response and injury. *Curr Opin Crit Care* 2013; 19:123–127. 23448974 10.1097/MCC.0b013e32835eba6d

[33] Jimenez MF Marshall JC Source control in the management of sepsis. *Intensive Care Med* 2001; 27 Suppl 1:S49–62. 11307370 10.1007/pl00003797

[34] Kim JJ Tsukamoto MM Mathur AK Delayed paracentesis is associated with increased in-hospital mortality in patients with spontaneous bacterial peritonitis. *Am J Gastroenterol* 2014; 109:1436–1442. 25091061 10.1038/ajg.2014.212

[35] Marshall JC Principles of source control in the early management of sepsis. *Curr Infect Dis Rep* 2010; 12:345–353. 21308516 10.1007/s11908-010-0126-z

